# Synthesis of multi-shelled ZnO hollow microspheres and their improved photocatalytic activity

**DOI:** 10.1186/1556-276X-9-468

**Published:** 2014-09-04

**Authors:** Xiangyun Zeng, Jiao Yang, Liuxue Shi, Linjie Li, Meizhen Gao

**Affiliations:** 1 Key Laboratory for Magnetism and Magnetic Materials of Ministry of Education, Lanzhou University, 730000 Lanzhou, People’s Republic of China; 2 School of Physical Science and Technology, Lanzhou University, Lanzhou, Gansu 730000, People’s Republic of China

**Keywords:** ZnO, Carbonaceous microspheres, Hollow microspheres, Photocatalytic

## Abstract

Herein, we report an effective, facile, and low-cost route for preparing ZnO hollow microspheres with a controlled number of shells composed of small ZnO nanoparticles. The formation mechanism of multiple-shelled structures was investigated in detail. The number of shells is manipulated by using different diameters of carbonaceous microspheres. The products were characterized by X-ray powder diffraction, scanning electron microscopy, and transmission electron microscopy. The as-prepared ZnO hollow microspheres and ZnO nanoparticles were then used to study the degradation of methyl orange (MO) dye under ultraviolet (UV) light irradiation, and the triple-shelled ZnO hollow microspheres exhibit the best photocatalytic activity. This work is helpful to develop ZnO-based photocatalysts with high photocatalytic performance in addressing environmental protection issues, and it is also anticipated to other multiple-shelled metal oxide hollow microsphere structures.

## Background

With the sustainable development of industry and society, the contamination of the environment caused by organic pollutants is becoming an overwhelming problem all over the world [1]. In recent years, conventional biological, physical, and chemical treatment methods have been studied widely. Since the semiconductor-based photochemical electrode reported by Fujishima and Honda in 1972 [2], photoactive nanomaterials as photocatalysts, especially semiconductor nanomaterials, have attracted the most attention to the degradation of organic compounds for the purpose of purifying wastewater. This is due to their high photocatalytic activity and excellent chemical and mechanical stability, and it is also an easy way to utilize the energy of solar light, abundantly available everywhere in the world [3]. Naturally, it is of substantial importance to carry out works related to semiconductor photocatalysis. Among these wide-bandgap semiconductors used in photoelectrochemical and photocatalytic applications, ZnO plays an important role in degrading various organic pollutants and photodegradation of bacteria due to its high catalytic activity, low cost, and environmental friendliness [4-6]. However, ZnO is a semiconductor with a bandgap of 3.37 eV and a large exciton binding energy of 60 meV at room temperature which results in the poor utilization of sunlight, limiting its photocatalytic efficiency. Therefore, it is essential to improve the photocatalytic properties of ZnO. According to the principle of photocatalysis, much research focuses on enhancing the surface areas of semiconductor nanomaterials by developing nanoscaled or porous appearance because a large surface area can achieve stronger light harvesting and provide more active sites at which the photocatalytic reaction occurs, small nanoparticles shorten the distance that electrons and holes migrate from bulk to reaction active sites to lower the possibility of recombination of the photogenerated charges, and the porosity can improve the photon application efficiency [7]. Thus, a number of efforts have been attracted to obtain high catalytic activity by manufacturing different ZnO nanostructures, such as nanoneedles [8], nanowires [9,10], nanorods [11,12], nanotetrapods [13], nanoplatelets [14], nanotubes [15,16], nanotowers [17], nanoflowers [18-20], and hollow nanospheres [21,22].

Hollow spheres and hollow spheres with multiple shells are of great interest in many current and emerging areas of technology because their unique structures enable physical properties such as uniform size, low density, and large surface area, which make them attractive materials for applications, such as sensors [23-26], catalysis [27-29], drug delivery [30-34], energy conversion [35-39], and storage systems [40-43]. For example, studies have demonstrated that multiple-shelled α-Fe_2_O_3_ hollow spheres are much more sensitive than α-Fe_2_O_3_ hollow spheres [44]. Multiple-shelled Co_3_O_4_ hollow microspheres were prepared and reported to have excellent cycle performance and enhanced lithium storage capacity [45]. Dong et al. reported the synthesis of quintuple-shelled SnO_2_ hollow microspheres, and the quintuple-shelled SnO_2_ hollow microspheres exhibited high-performance dye-sensitized solar cells [46]. Multiple-shelled metal oxides have been regarded as fascinating nanomaterials in the field of photocatalysis, partly due to their high quantum yield but also because the nanostructure gives a large surface-area-to-volume ratio and strong light-harvesting capabilities [47]. As a result, the study of the ZnO hollow nanosphere and hollow spheres with multiple shells of photocatalyst is both important and interesting.

Herein, we report a simple and general method to successfully fabricate ZnO hollow microspheres with a controlled number of shells by using carbonaceous saccharide microspheres with different diameters as templates. The photocatalytic property of the as-synthesized products is investigated by studying the degradation of methyl orange (MO) dye, and the triple-shelled ZnO hollow spheres with high surface area were proven to have excellent photocatalytic activity. The mechanism of formation of multiple-shelled ZnO hollow spheres and the reason for the high photocatalytic activity were also investigated.

## Methods

All chemicals were of reagent grade and were used as raw materials without further purification.

### Synthesis

In this work, carbonaceous saccharide microspheres were used as sacrificial templates and zinc nitrate hexahydrate (Zn (NO_3_)_2_·6H_2_O) were used as metal precursors. Taking the synthesis of single-shelled ZnO hollow microspheres as an example, the typical synthesis is described as follows. Carbonaceous microspheres were synthesized through the emulsion polymerization reaction of sugar under hydrothermal conditions as described elsewhere [48,49]. The diameter of the obtained carbon spherules could be controlled through regulating the concentration of the sugar solution and reaction time. The carbonaceous saccharide microspheres were washed several times with absolute ethanol and deionized water until the filtrate was clear. Newly prepared carbonaceous microspheres (0.5 g) with diameters about 500 nm were dispersed in 1.5 M zinc nitrate solution (water/ethanol = 1:3, *v*/*v*, 25 mL) with the aid of ultrasonication. After ultrasonic dispersion for 0.5 h, the resulting suspension was aged for 8 h at 60°C in a water bath, vacuum filtered, washed with deionized water for several times, and then dried at 80°C in an oven for 12 h. In order to remove the templates, the resulting black composite microspheres were heated to 350°C for 1 h and then the temperature was raised to 450°C in air at the rate of 1°C min^−1^ and kept at 450°C for 2 h. The single-shelled ZnO hollow microspheres were prepared as a white powder product after the tube furnace cooled down to room temperature naturally. The synthesis processes of double- and triple-shelled ZnO hollow microspheres were also synthesized by following a similar procedure. The ZnO nanoparticles were synthesized by combustion method. In brief, 3 g Zn (CH_3_COO)_2_ 2H_2_O and 1 g CO (NH_2_)_2_ were dissolved into 5 mL deionized water, and then the NH_3_ H_2_O was dropwise added into the solution until the solution turned into highly viscous gel precursors. Then the obtained viscous gel precursors were heated quickly at 500°C, and the precursors spontaneously ignited to produce white ZnO powders.

### Characterization

The crystal phase, morphology, and composition of the produced products were characterized by X-ray powder diffraction (XRD; Rigaku RINT2400, Rigaku, Tokyo, Japan) with Cu Kα radiation (*s* = 1.5418 Å), field-emission scanning electron microscopy (FE-SEM; Hitachi S-4800, Hitachi, Tokyo, Japan), and transmission electron microscopy (TEM; FEI Tecnai G2 F30, FEI, Oregon, USA). The Fourier transform infrared (FTIR) spectrum of the precursor was recorded between 400 and 4,000 cm^−1^ on a Nicolet NEXUS 670 FTIR spectrometer (Thermo Fisher Scientific, Massachusetts, USA). Thermogravimetric analysis (TGA) was carried out in air at a heating rate of 1.00°C min^−1^ from 35.00°C to 700.00°C using a PerkinElmer Diamond TG/DTA instrument (PerkinElmer, Massachusetts, USA). Diffuse reflectance absorption spectra (DRS) were measured by a PerkinElmer 950 UV–vis spectrophotometer equipment (PerkinElmer, Massachusetts, USA), and the recorded range of the spectra is 250 to 800 nm.

Photoelectrochemical characterizations were carried out in a three-electrode system with the aid of the electrochemical workstation CHI 660 (CH Instruments, Texas, USA) and a conventional three-electrode system. Indium tin oxide covered by a thin film of the samples was used for the working electrode, with an active area of 1 cm^2^. Platinum and Ag/AgCl (saturated KCl) electrode were used as the working electrode, auxiliary electrode, and reference electrode, respectively.

MO decomposition tests were carried out to study the photocatalytic activities of the as-synthesized products. Typically, 20 mg of photocatalysts was added into 300 mL of aqueous solution of the MO dyes (10 mg L^−1^), and the solution was simultaneously sonicated and shaken for 10 min in an ultrasonic cleaning bath. Prior to the irradiation, the suspensions were magnetically stirred in the dark for 30 min to realize adsorption equilibrium; afterward, the photoreaction vessel was exposed to UV irradiation (500-W high-pressure Hg lamp with the main wavelength at 365 nm) under ambient conditions to start the photocatalytic reaction. At given time intervals, a certain volume of suspension (approximately 3 mL) was withdrawn. With the help of centrifugation, the catalyst was recovered and the MO concentration of the sample at different intervals was monitored using a spectrophotometer (WFJ-7200, Unico, Franksville, WI, USA). The cycling runs were carried out to demonstrate the stability and reusability of triple-shelled ZnO hollow microspheres. After one cycle, the photocatalyst was filtrated and washed drastically with deionized water, and then the succeeding cycling runs are the same as the first cycle.

## Results and discussion

**Figure 1:**
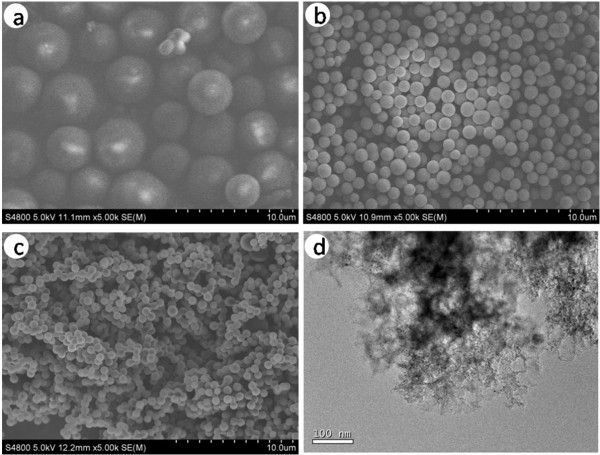
**SEM images of monodispersed carbonaceous templates and TEM image of the as-prepared carbon spherules. (a-c)** SEM images of monodispersed carbonaceous templates prepared under different glucose concentrations: **(a)** 1 mol L^−1^, **(b)** 0.5 mol L^−1^, **(c)** 0.25 mol L^−1^. **(d)** TEM image of the as-prepared carbon spherules.

Figure 1a, b, c shows the SEM images of the monodispersed carbonaceous templates with three sizes prepared using the hydrothermal method at 200°C under different concentrations of sugar solution from 0.25 to 1 mol L^−1^. A perfect sphere shape, a uniform diameter size, and monodispersed spherules can be seen. The diameter of the monodispersed carbon spherules varies from 0.5 to 4 μm. It indicates that the concentrations of the sugar solution play an important role in the diameter of the spherules. Figure 1d shows a TEM image of the fragment of carbon spherules. Clearly, there are large quantities of micropores of the carbonaceous templates. The key to the formation of the micropores is the escape of water through the flexible dewatering sugar in the experiment. Energy-dispersive X-ray spectroscopy (EDS) (Additional file 1: Figure S1) shows that the carbon spherules consisted of C and O elements. The FTIR spectrum of the carbon spherules was characterized as shown in Additional file 1: Figure S2. The strong absorption in the intensity of the bands at 3,433 cm^−1^ is the characteristic of the O-H stretching mode. The absorption bands lying at 1,703 and 1,625 cm^−1^ are associated with the stretching of the C = O and C = C groups, respectively. The absorption band at 1,000 to 1,300 cm^−1^ indicates the appearance of the stretching of C-OH. The FTIR spectra provide a clear evidence that the carbon spherules are rich with surface functional groups, which play an important role in the adsorption of the metal ion [19].

In addition, the precursor is characterized by TGA. Additional file 1: Figure S3 shows that there is a sharp mass loss from room temperature to 250°C in air, indicating that the start temperature of the decomposition of the precursor is around 250°C. The results of the TGA suggest that the carbon template could be completely removed. According to the TGA curve, we obtained the ZnO multi-shelled hollow spheres by choosing a temperature of 450°C for the thermal treatment of the precursor to ensure its complete decomposition.

**Figure 2:**
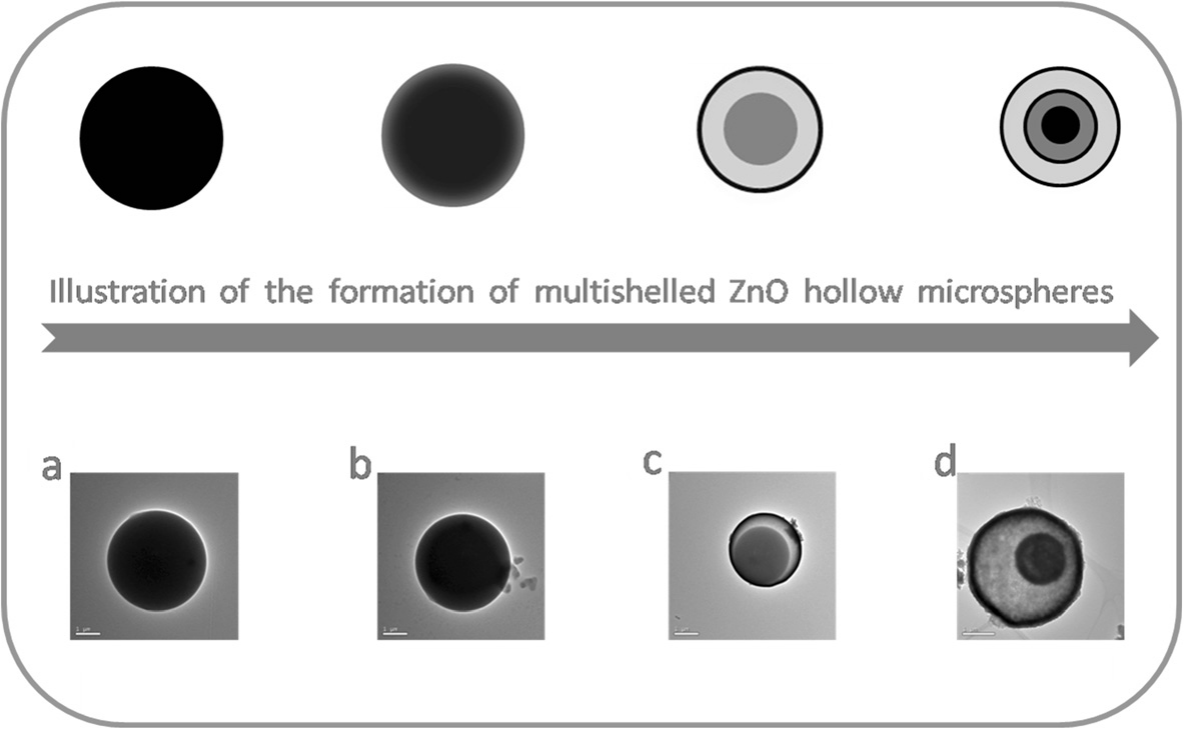
**Schematic illustration and TEM images of the formation of multiple-shelled ZnO hollow microspheres. (a)** 0 h. **(b)** 0.5 h. **(c)** 1 h. **(d)** 2 h.

**Figure 3:**
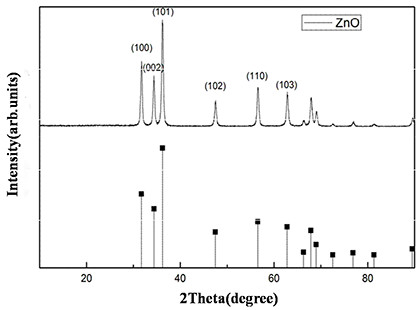
XRD pattern of the double-shelled ZnO hollow microspheres.

The evolution process of shell formation was obtained by carrying out the reactions at the same temperatures for various times. The carbonaceous microspheres are full of zinc ions, heated to 450°C at the rate of 1°C min^−1^. The TEM images of the products were obtained after different holding times in the heating process. After heating to 450°C for 0.5 h, the carbonaceous microspheres have no obvious change. When the holding time increases to 1 h, a shell around a solid sphere was formed. As testified by the XRD and EDS data, the resultant materials were obtained without carbon and had a triple-shelled hollow structure. Figure 2 also illustrates the general process of fabricating triple-shelled hollow zinc oxide microspheres. In order to increase the surface functional groups of carbonaceous microspheres, such as hydroxyl or carboxylic acid, which are important and necessary for the adsorption of zinc ions, the carbonaceous microspheres were immersed in 1 mol L^−1^ aqueous HCl for 24 h. Because of the differential shrinking rate of carbonaceous particles and zinc precursors, multiple-shelled ZnO hollow spheres were generated. The amount of metal ion adsorbed by the carbonaceous microspheres is the crucial factor, which determines the number of shells of hollow spheres. It is easy to understand that the carbonaceous microspheres are larger and much more zinc ions are absorbed inside their inner core, which lead to hollow spheres with more shells. Based on this, it is feasible to control the number of shells of ZnO hollow microspheres by using the carbonaceous microsphere temples with different diameters.The crystalline structures of the prepared ZnO hollow microspheres were investigated by XRD. From Figure 3, it can be seen that the XRD pattern of the obtained ZnO hollow microspheres has identical peaks, which is consistent with wurtzite-structured ZnO (JCPDS Card No. 36–1451) without any other phases present, indicating the formation of pure zinc oxide.

**Figure 4:**
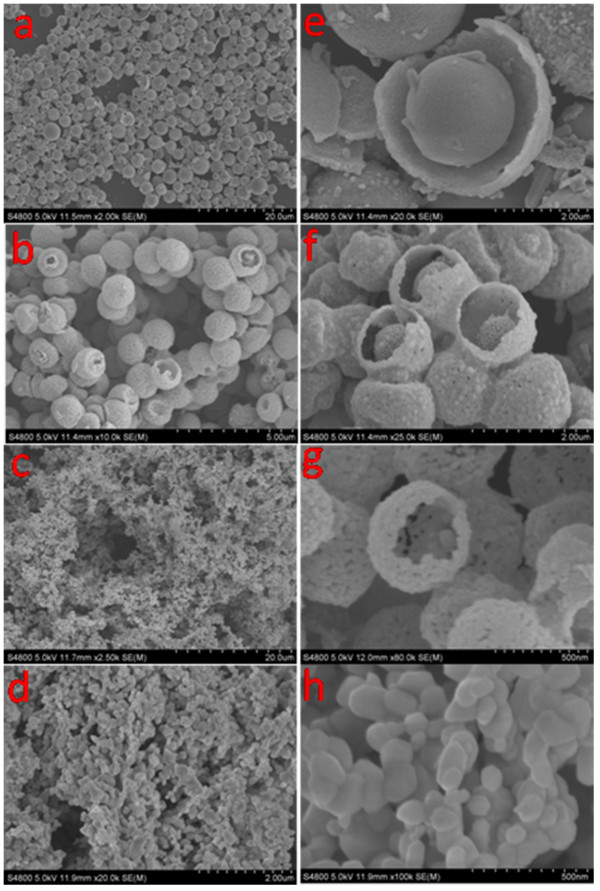
**Low- and high-magnification SEM images of the triple-, double-, and single-shelled ZnO hollow spheres and ZnO nanoparticles. (a, e)** Triple-, **(b, f)** double-, **(c, g)** and single-shelled ZnO hollow spheres and **(d, h)** ZnO nanoparticles.

**Figure 5:**
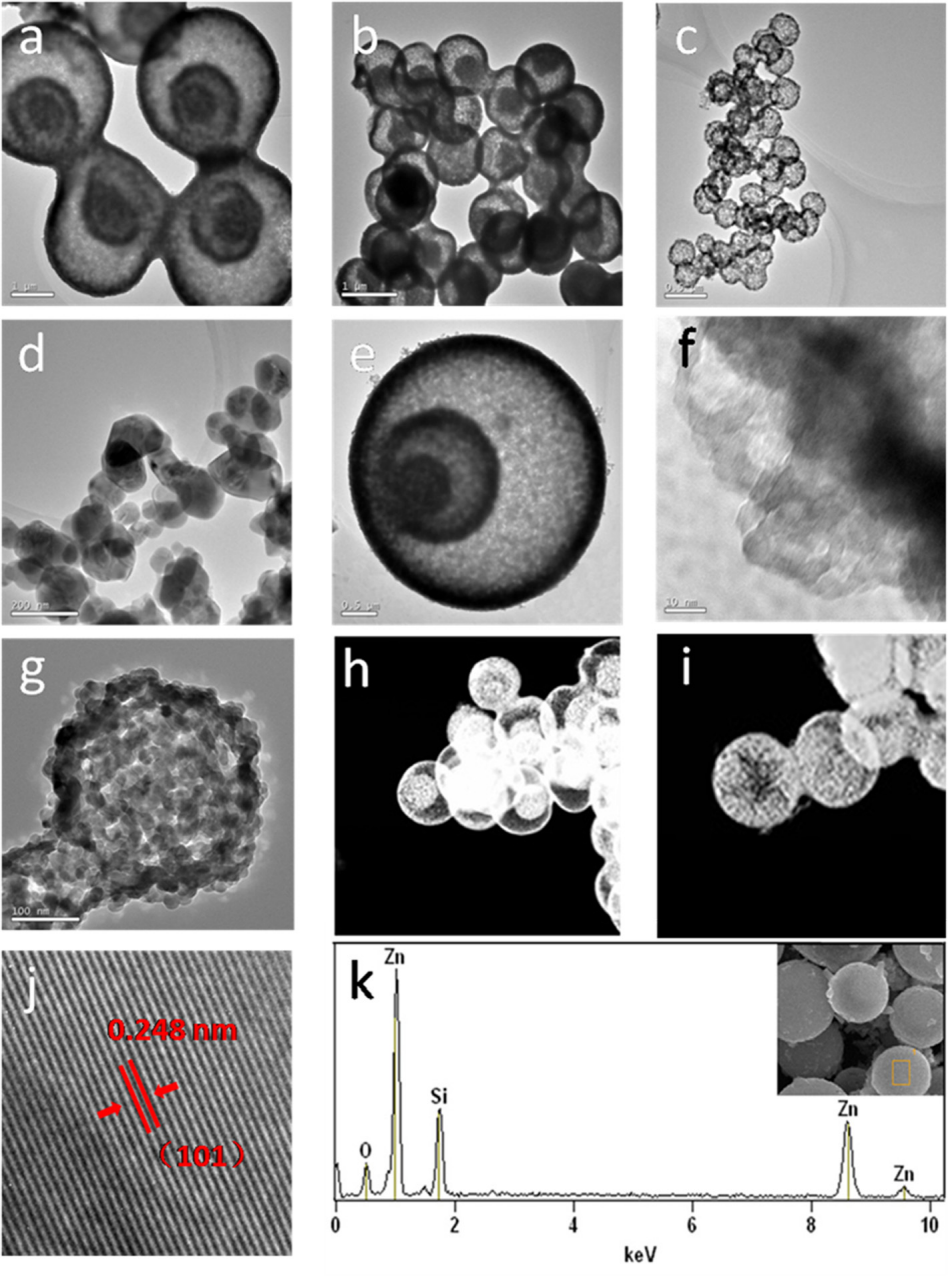
**TEM, HRTEM, and STEM images and EDS spectrum of ZnO hollow spheres. (a-d)** TEM images of the triple-, double-, and single-shelled ZnO hollow spheres and ZnO nanoparticles. **(e-g)** High-magnification TEM images of the triple- and single-shelled ZnO hollow spheres. **(h, i)** STEM images of the triple-, double-, and single-shelled ZnO hollow spheres. **(j, k)** HRTEM image and EDS spectrum of an individual triple-shelled ZnO hollow microsphere. The inset of Figure 5k is the SEM image of the sample, and the place marked by a rectangle shows the specific position of EDS analysis.

Figure 4 and Additional file 1: Figure S4 show the morphology and size distribution of the ZnO hollow spheres and nanoparticles. It is easy to discover the average values and standard deviations of the diameters of ZnO hollow spheres and nanoparticles. It can be seen that the diameter and number of shells of the as-prepared ZnO hollow spheres change when different carbonaceous saccharide microspheres are used. From the TEM (Figure 5a, b, c and Additional file 1: Figure S5) and scanning TEM (STEM) (Figure 5h, i) images, it can be clearly seen that the spheres are with triple, double, and single shells. Some hollow spheres were broken, and the cross sections of the ZnO hollow spheres also show the number of ZnO hollow spheres (Figure 4e, f, g). The diameter of the ZnO hollow spheres is in the range of 0.5 to 4 μm and the number of shells in the range of 1 to 3. Figures 4d, h and 5d display SEM and TEM images of the as-prepared ZnO nanoparticles. Figure 5f, g shows a high-resolution TEM (HRTEM) image recorded from the outer shell of one hollow sphere and single-shelled hollow sphere. It can be observed that the shell is porous and composed of crystalline nanoparticles. Figure 5j shows the HRTEM image of triple-shelled hollow nanospheres, from which we determined the interplanar distances to be 0.248 nm, which corresponds well to the lattice spacing of the (101) plane of wurtzite ZnO. Figure 5k reveals that these nanostructures are mainly composed of the elements Zn and O, indicating that pure ZnO nanospheres were obtained, and the elemental mapping of ZnO hollow spheres also shows the composition and element distribution of the ZnO hollow spheres. The result is in accord with the XRD data.

**Figure 6:**
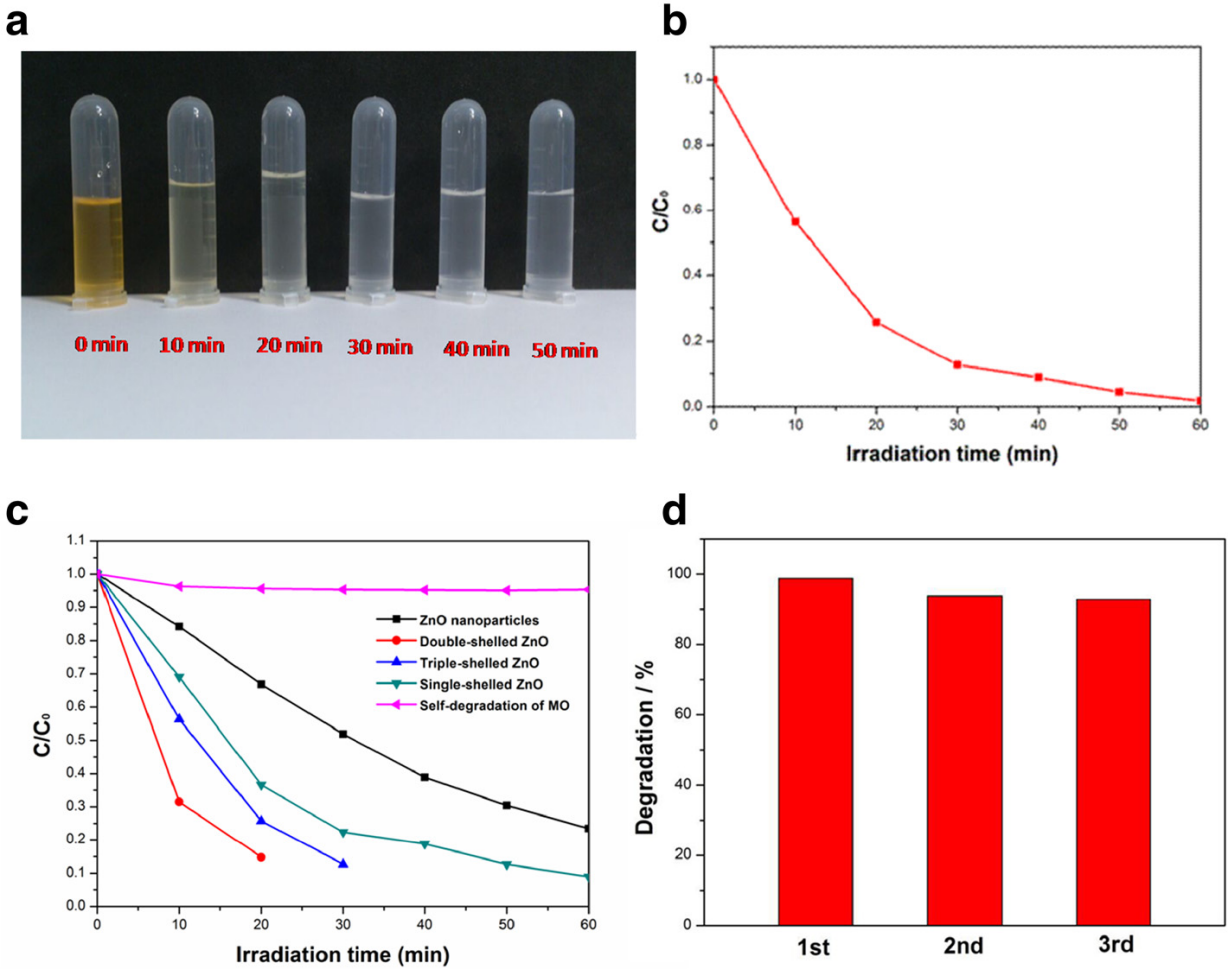
**Photographs, concentrations, and photocatalytic degradation of MO. (a)** Photographs of MO irradiated by UV light for different periods of time. **(b)** Concentration of MO in the solution with the triple-shelled ZnO hollow spheres. **(c)** Concentration of MO in the solution with different photocatalysts versus the 30-min exposure time to UV irradiation. **(d)** Cycling runs in the photocatalytic degradation of MO in the presence of the triple-shelled ZnO hollow spheres.

In order to testify the prediction that multiple-shelled hollow microsphere architectures can provide even more efficient multi-reflections of UV light within their interiors compared with nanoparticles and single-shelled and double-shelled hollow microspheres, the photocatalytic activities of the triple-shelled, double-shelled, and single-shelled ZnO hollow spheres and ZnO nanoparticles were measured and the photocatalytic decomposition of MO was carried out in a vessel containing a suspension of 20 mg in 500 mL of MO solution (10 mgL^−1^) under UV light irradiation (500 W of the UV lamp). Before the irradiation, the suspensions were magnetically stirred in the dark for 30 min to ensure adsorption/desorption equilibrium. Photographs of MO under UV light irradiation for different periods of time are shown in Figure 6a. The triple-shelled ZnO hollow spheres behaved as effective catalysts in the photodegradation of MO (Figure 6b), and it can be seen that the MO solution was almost degraded completely within 30 min. Figure 6c shows the degradation rate of MO over different photocatalysts; ZnO nanoparticles (as shown in Figure 5d, e, f, g, h) exhibit the lowest activity, while triple-shelled ZnO hollow microspheres (as shown in Figure 5a, b, c, d, e) show the highest activity after irradiation for the same time. As expected, the triple-shelled ZnO hollow microspheres exhibit the best photodegradation behavior. Since all the samples possess similar crystalline structures, such an improvement of photocatalytic activity is governed by the unique multiple-shelled hollow morphology.

**Figure 7:**
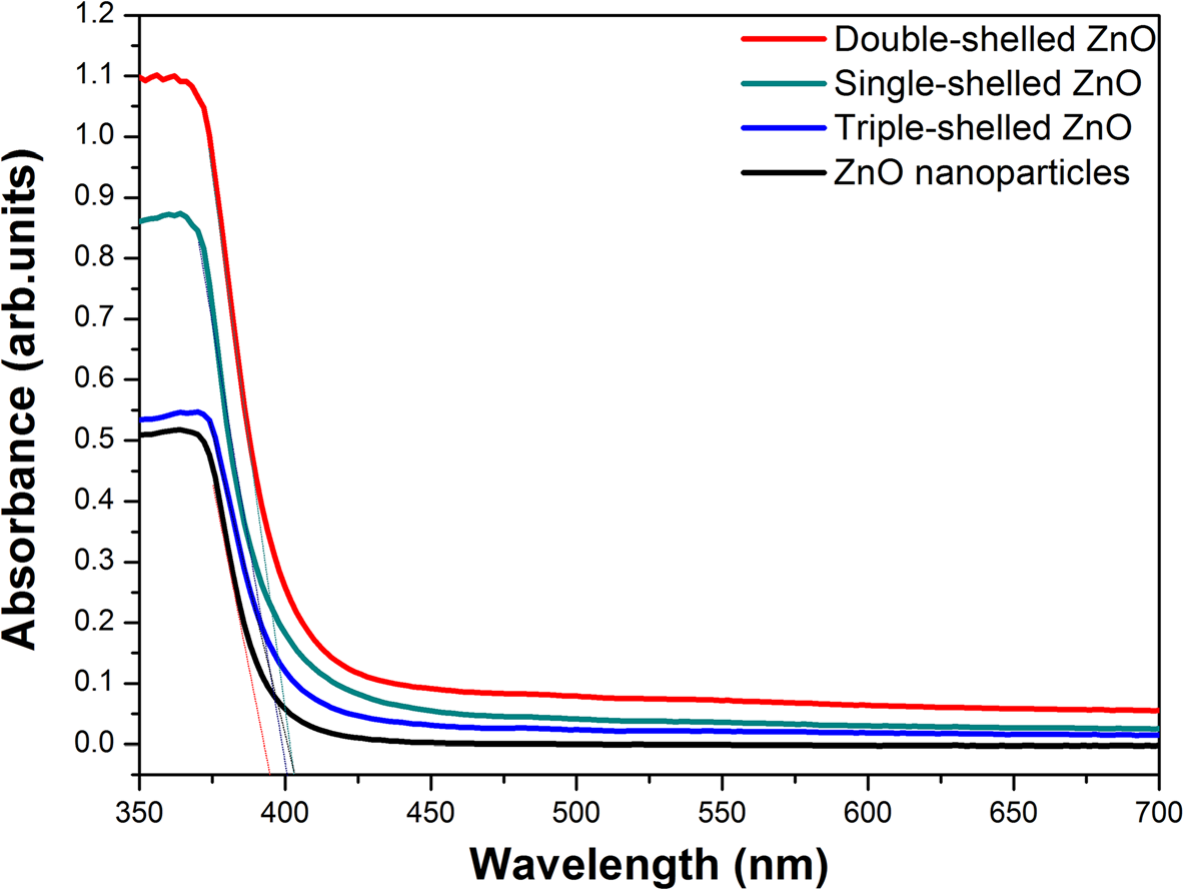
The room-temperature UV–vis DRS of ZnO nanoparticles and the triple-, double-, and single-shelled ZnO hollow spheres.

**Figure 8:**
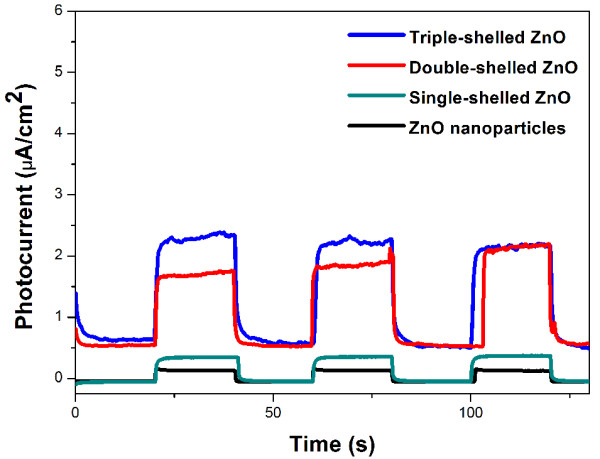
Photocurrent response of the ZnO nanoparticles and the triple-, double-, and single-shelled ZnO hollow spheres.

**Figure 9:**
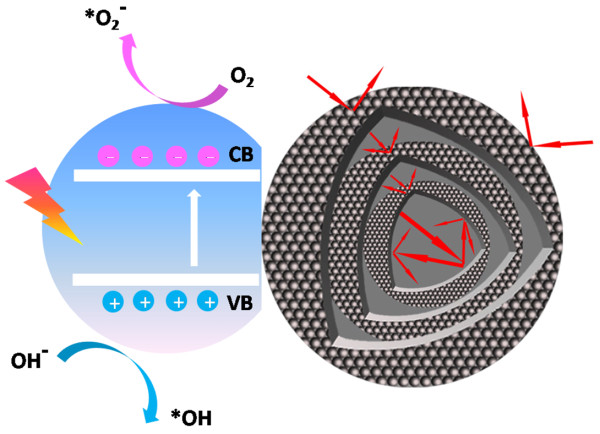
Schematic diagrams showing the photocatalytic performance of the triple-shelled ZnO hollow spheres.

Figure 7 shows the room-temperature UV–vis DRS of four samples. The bandgap energies (Eg) calculated on the basis of the corresponding absorption edges are 3.14 eV (ZnO nanoparticles) and 3.08 eV (triple-shelled ZnO). Compared with ZnO nanoparticles, the ZnO hollow microspheres show redshifts of the absorption edge, and it is found that the wavelength for absorption edge increases with increasing number of the shell of ZnO hollow microspheres. The results may come from the raising of the base absorption line which mainly contributed to the diameter of the nanoparticles of ZnO hollow microspheres being less than the diameter of ZnO nanoparticles [7]. All the absorption edge of these samples is still in the UV light region. Therefore, the photocatalytic activities of the samples were measured using the Hg lamp with the main wavelength at 365 nm as the irradiation source.In order to further demonstrate this structure–property relationship, we fabricated film photodetector devices composed of the ZnO nanoparticles and the triple-, double-, and single-shelled ZnO hollow spheres and measured their reproducible responses to on-off light cycles, and the results are shown in Figure 8. It demonstrates that the photodetector devices composed of the triple-shelled ZnO hollow microspheres have a very fast response time and excellent repeatability. It was found that the photocurrent on the triple-shelled ZnO hollow microspheres is about 6.5 times higher than that on the ZnO nanoparticles. It indicated that the multiple-shelled ZnO hollow microspheres can be used in UV light photodetectors.According to the above experimental results and analysis, the reasons for the optimized photocatalytic activity and enhancement of the photocurrent for triple-shelled ZnO hollow microspheres were discussed in detail and the photocatalytic mechanism was proposed. The mechanism for degradation of MO caused by the triple-shelled ZnO hollow microspheres under UV light irradiation is shown in Figure 9. This can be ascribed to the following. Firstly, the triple-shelled hollow microsphere structures can enable multiple light reflection and scattering between the outer spherical shell and the two interior shells compared with the nanoparticles and single-shelled hollow spheres to provide a more efficient way to enhance light-harvesting efficiency. (The insets show a schematic illustration of Figure 6c). Secondly, this structure also can supply more specific surface area for adsorbing more dye molecules which contribute to the high degradation performance of the triple-shelled hollow spheres. Furthermore, the better photo-induced charge separation efficiency of triple-shelled hollow microsphere structures plays an important role in a significant enhancement in conductivity. Therefore, the triple-shelled ZnO hollow microsphere structures which are in favor of these features have much better photocatalytic activity than the ZnO nanoparticles and ZnO hollow spheres.

## Conclusions

In summary, it has been demonstrated that different numbers of shells of ZnO hollow microspheres can be successfully prepared by a facile route. Compared with nanoparticles and single-shelled hollow spheres, the obviously improved photocurrent responses and enhanced photocatalytic activity of the triple-shelled hollow microspheres have been achieved. The reason for this is attributed to the multiple-shelled hollow microsphere structure that captures large numbers of ultraviolet light photons and has large surface areas for absorbing more dye molecules. The multiple-shelled ZnO hollow nanospheres can be regarded as an excellent photocatalyst candidate and may be used in other fields such as UV and gas sensors and solar cells. This new synthetic concept is also helpful to controllably construct other multiple-shelled metal oxide hollow microsphere structures with enhanced properties for microelectronics, optoelectronics, and other applications.

## Abbreviations

DRS: diffuse reflectance absorption spectra; FE-SEM: field-emission scanning electron microscopy; FTIR: Fourier transform infrared spectrum; MO: methyl orange; TEM: transmission electron microscopy; TGA: thermogravimetric analysis; UV: ultraviolet; XRD: X-ray powder diffraction.

## Competing interests

The authors declare that they have no competing interests.

## Authors’ contributions

The experiments and characterization presented in this work were carried out by XZ, JY, LS, and LL. The experiments were designed by XZ and MG. XZ, JY, and MG analyzed and discussed the results obtained from the experiments. The manuscript was prepared by XZ. MG helped with the draft editing. All authors read and approved the final manuscript.

## Supplementary Material

Additional file 1 **Supporting information. Figure S1**. Energy-dispersive spectroscopy characterization of the carbon spherules. **Figure S2**. FTIR spectrum of the carbon spherules. **Figure S3**. TGA curve of the precursor. **Figure S4**. Low-magnification SEM images of the triple-, double-, and single-shelled ZnO hollow spheres and ZnO nanoparticles. **Figure S5**. High-magnification TEM images of the triple-, double-, and single-shelled ZnO hollow spheres and ZnO nanoparticles. Figure S6. Elemental mapping of the ZnO hollow spheres. Click here for file
