# Supplementary figures and images for: Synthesis of multi-shelled ZnO hollow microspheres and their improved photocatalytic activity
Source: Nanoscale Res Lett. 2014 Sep 4;9(1):468. doi: 10.1186/1556-276X-9-468 (PMC4200476; doi:10.1186/1556-276X-9-468)

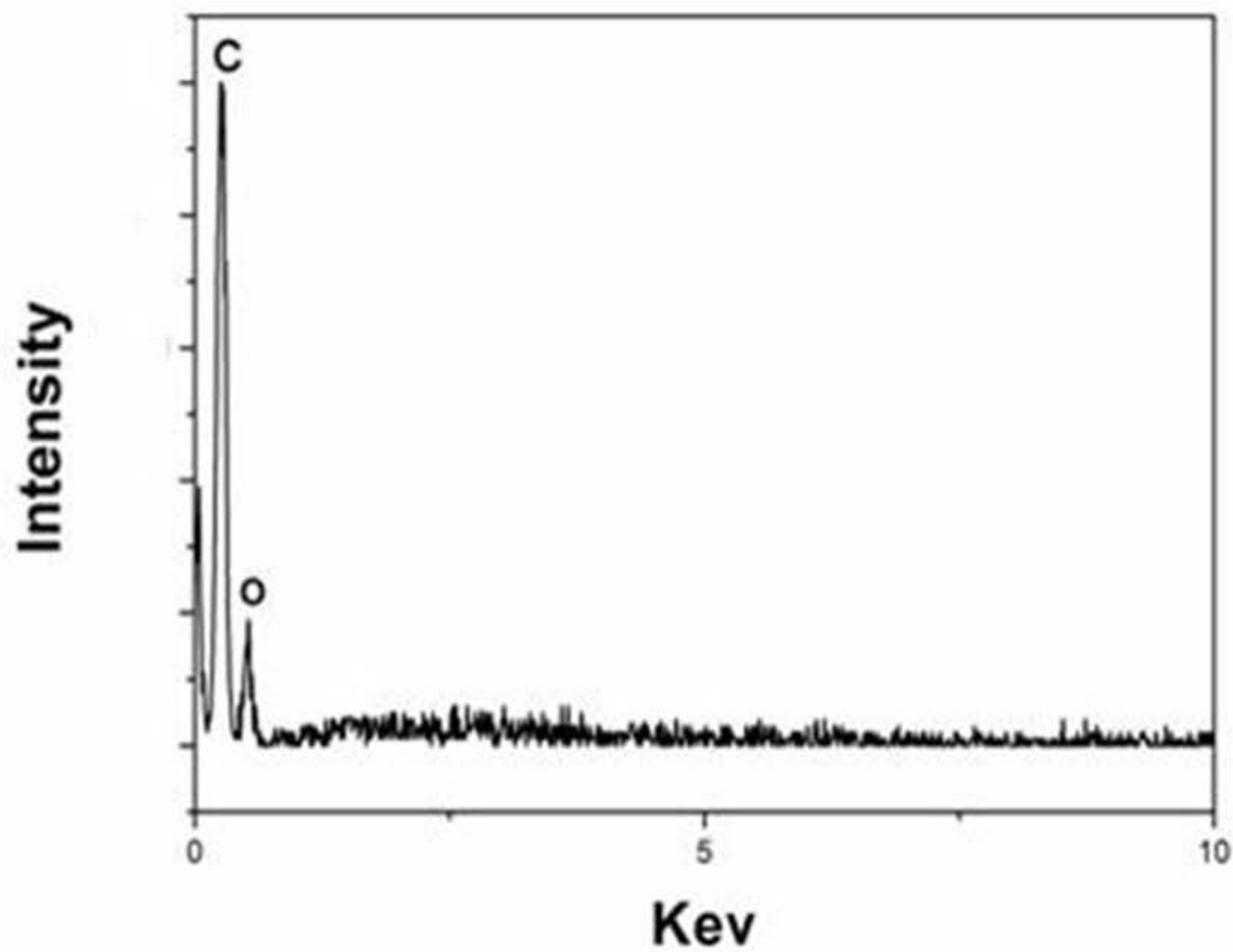

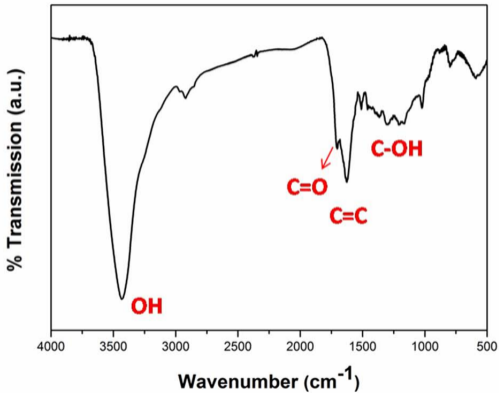

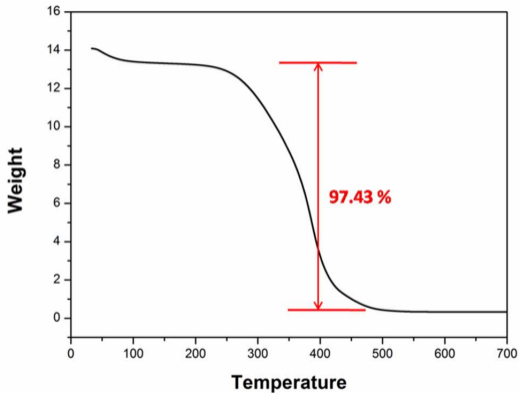

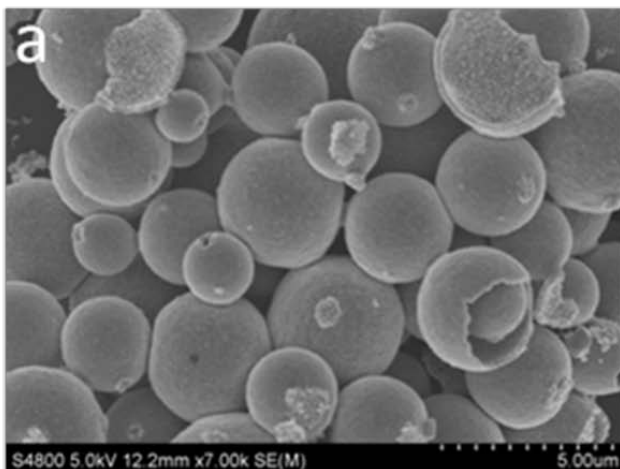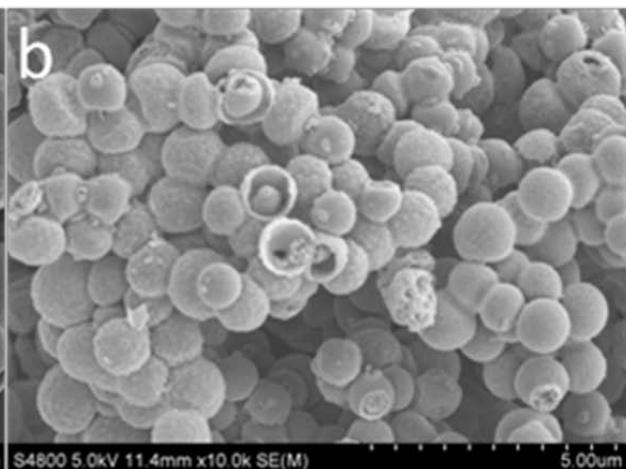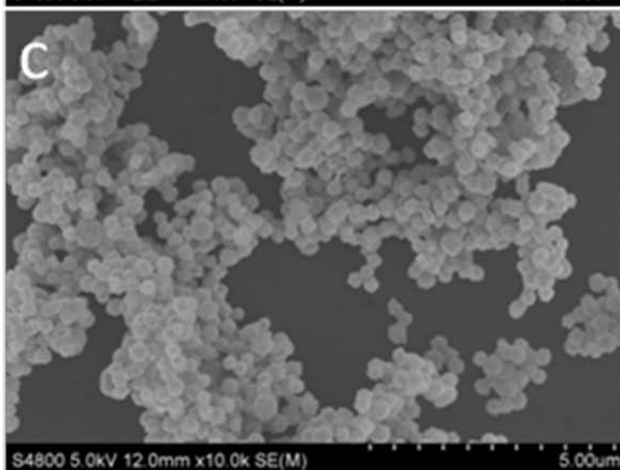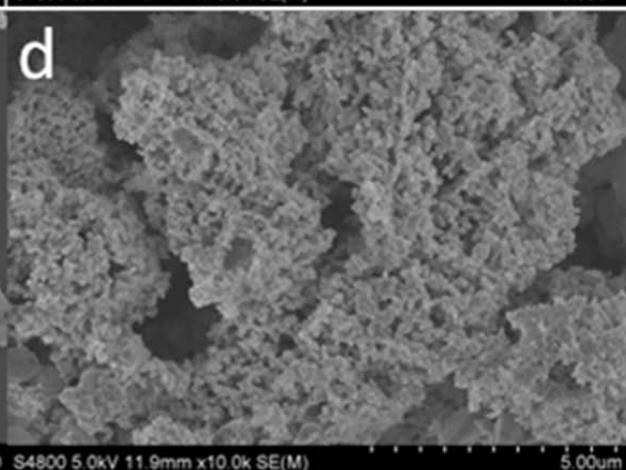

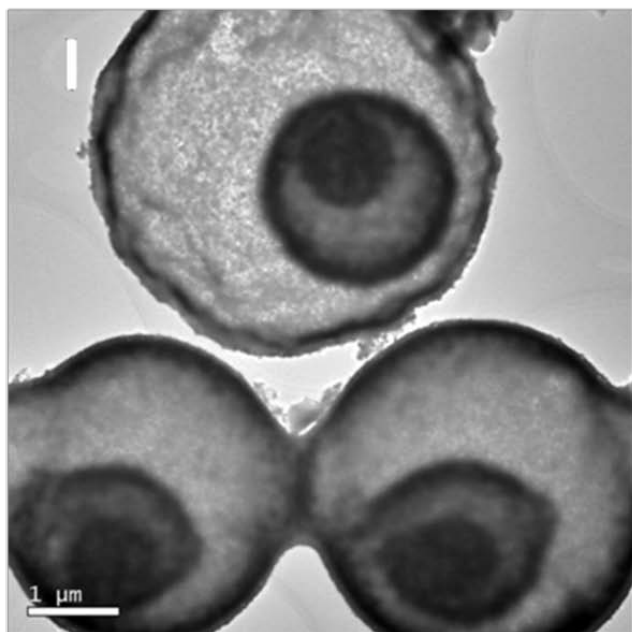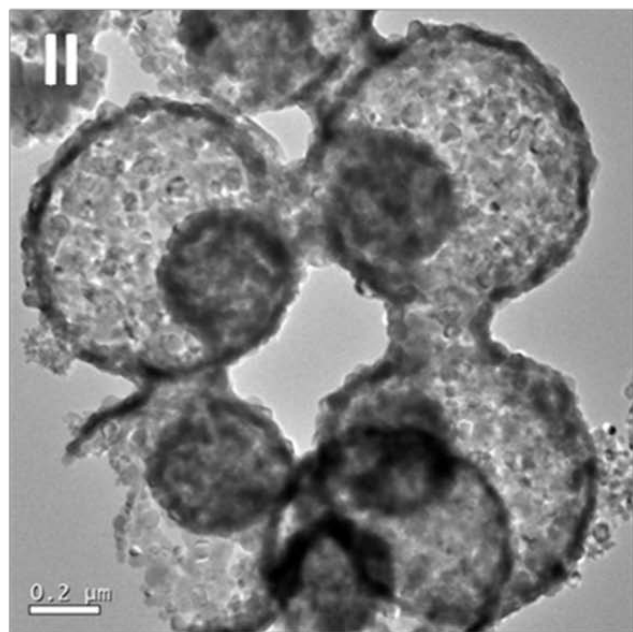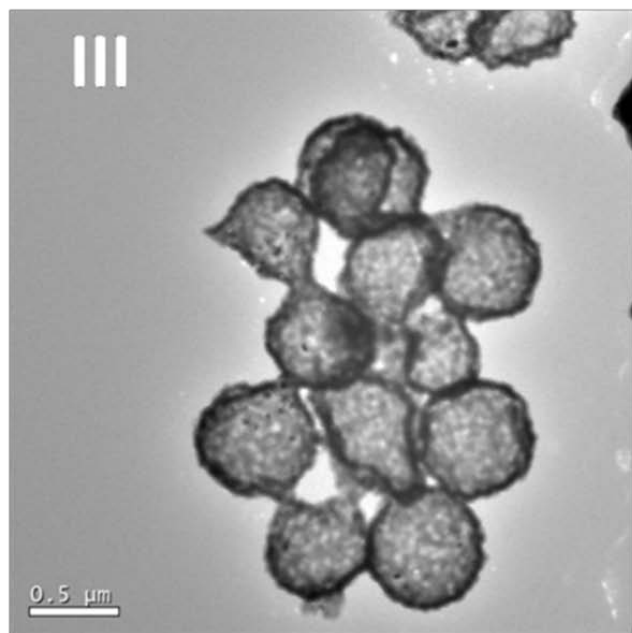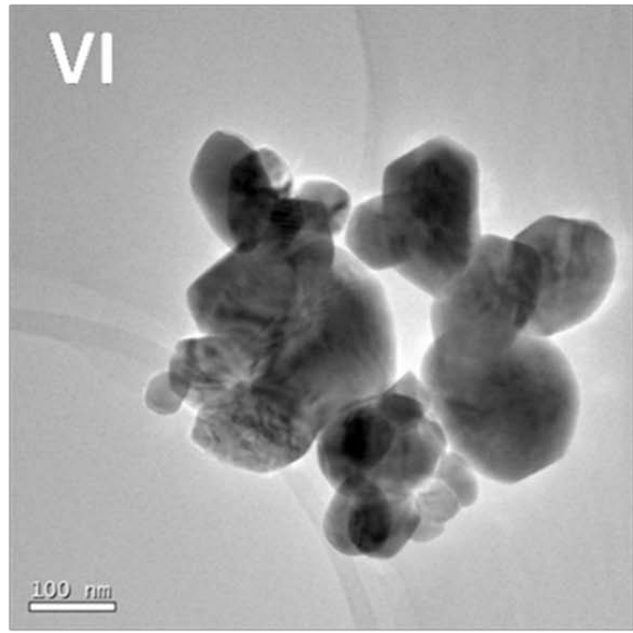

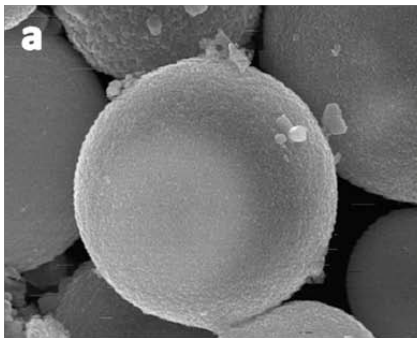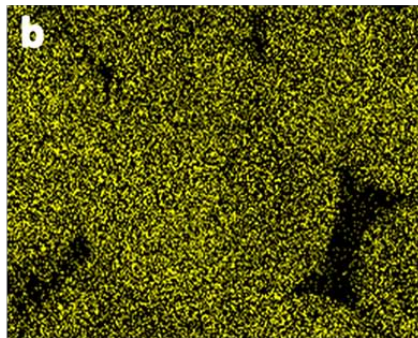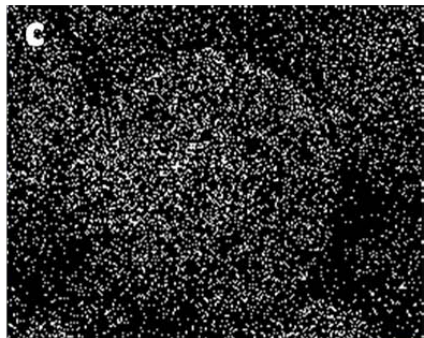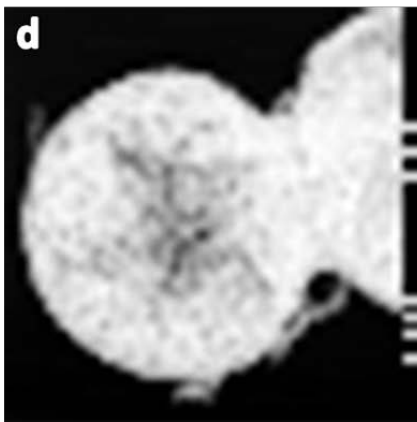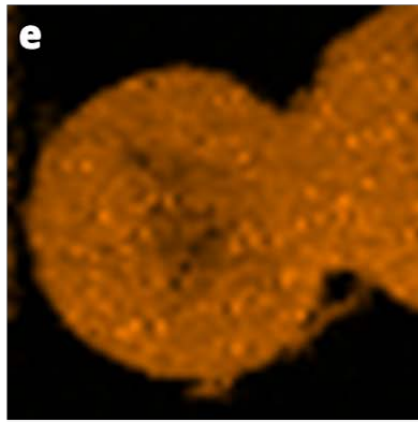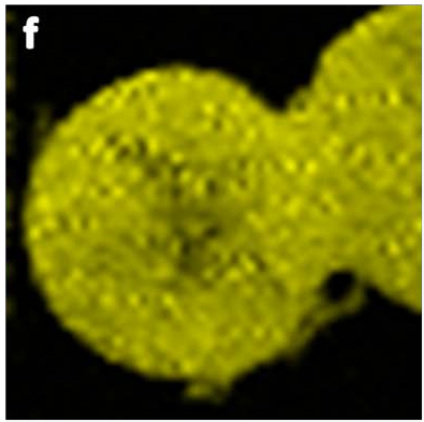

Supplement: Additional file 1 — Supporting information. Figure S1. Energy-dispersive spectroscopy characterization of the carbon spherules. Figure S2. FTIR spectrum of the carbon spherules. Figure S3. TGA curve of the precursor. Figure S4. Low-magnification SEM images of the triple-, double-, and single-shelled ZnO hollow spheres and ZnO nanoparticles. Figure S5. High-magnification TEM images of the triple-, double-, and single-shelled ZnO hollow spheres and ZnO nanoparticles. Figure S6. Elemental mapping of the ZnO hollow spheres. [file 1556-276X-9-468-S1.pdf]
